# Cannabinoids as therapeutic agents in cancer: current status and future implications

**DOI:** 10.18632/oncotarget.2233

**Published:** 2014-07-17

**Authors:** Bandana Chakravarti, Janani Ravi, Ramesh K. Ganju

**Affiliations:** ^1^ Molecular Medicine and Biotechnology Department, Sanjay Gandhi Post Graduate Institute of Medical Sciences, Lucknow, India; ^2^ Department of Pathology, The Ohio State University, Columbus, Ohio, USA

**Keywords:** Cannabinoid receptors, cannabinoid agonists, cancer, signaling

## Abstract

The pharmacological importance of cannabinoids has been in study for several years. Cannabinoids comprise of (a) the active compounds of the Cannabis sativa plant, (b) endogenous as well as (c) synthetic cannabinoids. Though cannabinoids are clinically used for anti-palliative effects, recent studies open a promising possibility as anti-cancer agents. They have been shown to possess anti-proliferative and anti-angiogenic effects *in vitro* as well as *in vivo* in different cancer models. Cannabinoids regulate key cell signaling pathways that are involved in cell survival, invasion, angiogenesis, metastasis, etc. There is more focus on CB1 and CB2, the two cannabinoid receptors which are activated by most of the cannabinoids. In this review article, we will focus on a broad range of cannabinoids, their receptor dependent and receptor independent functional roles against various cancer types with respect to growth, metastasis, energy metabolism, immune environment, stemness and future perspectives in exploring new possible therapeutic opportunities.

## INTRODUCTION

*Cannabis sativa plant* has been used for several hundreds of years both recreationally and medicinally. Centuries ago, the Chinese medicine refers to cannabis plant for pain-relief and hallucination. It contains 3 major classes of bioactive molecules; flavanoids, terpenoids and more than 60 types of cannabinoids [1]. Cannabinoids are the active compounds of this marijuana plant. But, the use of cannabinoids is in question because of their phsychotropic and addictive issues. The most active constituent of this plant is Δ^9^-tetrahydrocannabinol (Δ^9^-THC), elucidated between 1940s and 1960s [2]. This discovery has opened the way to identification of the molecular action of various cannabinoids and the cannabinoid receptors. Evidence shows that smoking of cannabis preparations caused cancer of the respiratory and oral tracts or, at least, potentiated tobacco smoke-induced damages [3]. Cannabinoid is a family of complex chemicals (terpenophenolic compounds) that exert most of their actions by binding to and activating specific Gαi protein-coupled receptors named as cannabinoid receptor, CB1 (Central receptor) and CB2 (Peripheral receptor) respectively [4-5]. CB1 and CB2 have been cloned and characterized from mammalian tissues, the main difference between them being their tissue expression pattern [6]. CB1 receptors are ubiquitously located, with their highest presence found in the central nervous system (basal ganglia, hippocampus, cerebellum and cortex) where they mediate cannabinoid psychoactive effects [7-8]. CB1 receptors are also present in peripheral nerve terminals, as well as in extra-neural tissues such as testis, uterus, vascular endothelium, eye, spleen, ileum and in adipocytes [8]. CB2 receptor expression is mostly restricted to particular elements of the immune system (enriched area of B lymphocyte) [9-10]. The human CB2 receptor shows 68% amino acid homology with the CB1 receptor in the trans-membrane domains and a 44% overall homology [9]. Cannabinoid receptors and their endogenous ligands termed as the endocannabinoid system have been used as putative molecular targets for the treatment of various diseases, including neurodegenerative diseases (Alzheimer's disease, Parkinson's disease, Huntington's disease, etc.), neuropathic and inflammatory pain, glaucoma, multiple sclerosis, cardiovascular disorders and obesity etc [5].

Recently cannabinoid's role has been explored in the area of cancer research. Cancer is caused by uncontrolled proliferation of cells and the ability of these cells to invade into other tissues and spread. Anti-cancer agents function as apoptotic, cell cycle defective or DNA damage agents. A major discovery in cancer in cannabinoid use in cancer treatment is its ability in targeted killing of tumors. Several preclinical studies suggest that Δ^9^-THC, other naturally occurring cannabinoids, synthetic cannabinoid agonists and endocannabinoids have anti-cancer effects *in vitro* against lung carcinoma, gliomas, thyroid epithelioma, lymphoma, skin carcinoma, uterine carcinoma, breast cancer, prostate carcinoma, pancreatic cancer and neuroblastoma [4]. These findings were also supported by *in vivo* studies and the majority of effects of cannabinoids are mediated via CB1 and CB2. The transient receptor potential vanilloid type 1 (TRPV1) has been described as an additional receptor target for several cannabinoids. In addition, the palliative effects of cannabinoids include inhibition of nausea and emesis which are associated with chemo- or radiotherapy, appetite stimulation, pain relief, mood elevation and relief from insomnia in cancer patients. Synthetic THC (Marinol, Dronabinol) and its derivative nabilone (Cesamet), as well as Sativex, have been approved in several countries to control nausea and cancer-related pain in cancer patients undergoing chemotherapy [11-12]. In this review article we focused on the role of cannabionds in different cancer types and the respective signaling pathways.

### Cannabinoid and its receptor

**Fig.1:**
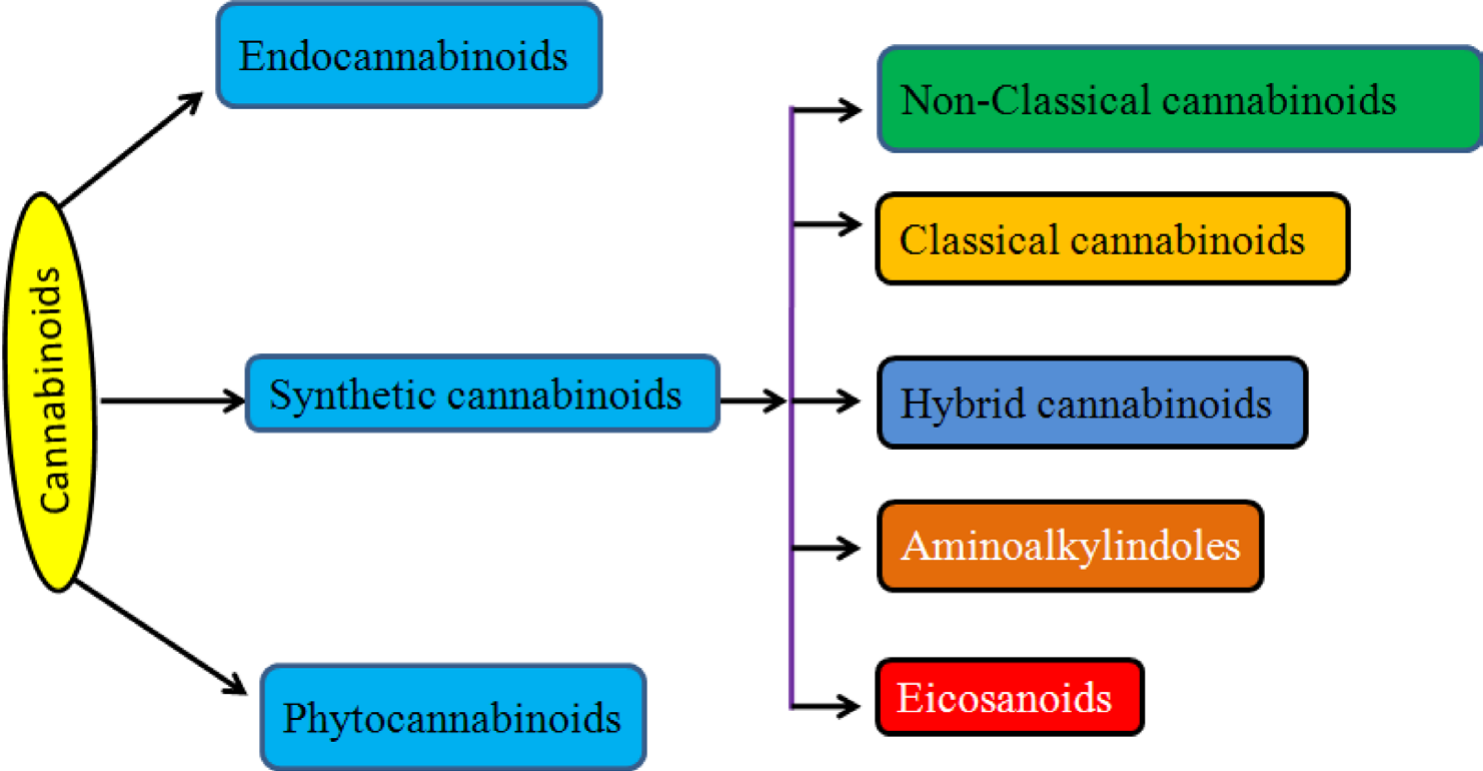
Cannabinoids and their classification This figure illustrates how cannabinoids are divided into three main categories according to their availability in nature.

Cannabinoids can be classified into three groups based on their source of production; endogenous cannabinoids (endocannabinoids), phytocannabinoids and synthetic cannabinoids (Fig.1) and their putative molecular targets (CB1 or CB2 receptor or TRPV1) have been identified (Table I). The central and most of the peripheral effects of cannabinoids rely on CB1 receptor activation.

**Table I:** Cannabinoid's structure and its role in different physiological processes

Cannabinoid's name (Abbreviation) and its target	Structure	Role
Anandamide (AEA) CB1 agonist	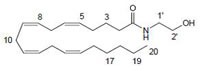	Analgesic, antiemetic, appetite stimulant, tumour growth inhibitor [159].
2-arachidonoyl-glycerol (2-AG) CB1/CB2 agonist	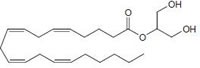	Analgesic, antiemetic, appetite stimulant, tumour growth inhibitor [159].
Palmitoyl-ethanolamide (PEA) CB2 agonist	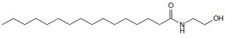	neuromodulatory and immunomodulatory [160].
Docosatetraenyl ethanolamide CB1 agonist	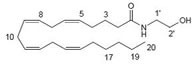	neuromodulatory and immunomodulatory [160].
Homo-γ-linoenylethanolamide CB1 agonist	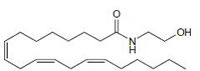	neuromodulatory and immunomodulatory [160].
Oleamide CB1 agonist	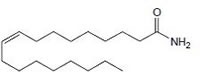	neuromodulatory and immunomodulatory [160].
Δ^9^-tetrahydrocannabinol (Δ^9^-THC) CB1/CB2 agonist	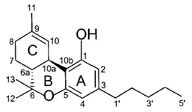	Analgesic, antiemetic, appetite stimulant tumour growth inhibitor [159].
Δ^8^-tetrahydrocannabinol (Δ^8^-THC) CB1/CB2 agonist	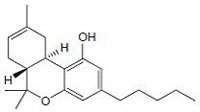	Anti-tumor agent, inhibitors of mitochondrial O2 consumption in human sperm, antiemetic, appetite stimulant [151, 161-163].
cannabidiol (CBD), CB1 agonist	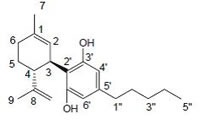	Anti-tumor agent, attenuate catalepsy, immunosuppressive, inflammatory or anti-inflammatory agent (depends upon used concentration of drug), antipsychotics [164-168]
Cannabigerol (CBG),	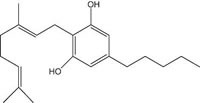	multiple sclerosis, antiemetic, anti-inflammatory agent, treatment for neurological disorder [169-171]
Cannabichromene (CBC),	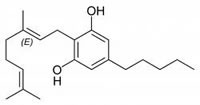	anti-inflammatory agent, treatment for neurological disorder, hypomotility, antinociception, catalepsy, and hypothermia [172-174]
Tetrahydrocannabivarin (THCV),	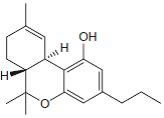	Hepatic ischaemia, anti-inflammatory [175-176]
Cannabigerovarin (CBGV),	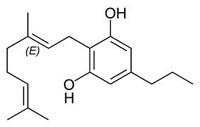	Anti-inflammatory [177]
HU-210 CB1/ CB2 Nonselective agonist	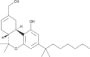	Analgesic, multiple sclerosis, neuroprotective [159]
CP-55,940 CB1/ CB2 Nonselective agonist		Anti-cancer agent, Analgesic, antiemetic, appetite stimulant
*R*-(+)-WIN 55,212-2 CB1/ CB2 Nonselective agonist	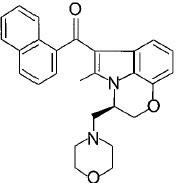	Analgesic, antiemetic, appetite stimulant, tumour growth inhibitor, multiple sclerosis [159]
JWH-015 CB2 selective agonist	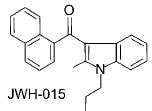	Anti-tumor,anti-inflammatory, antiemetic [178]
JWH-133 CB2 selective agonist	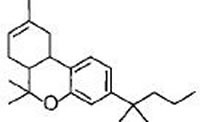	Neurological disorders, Anti-cancer [83, 179]
JWH-139 CB2 selective agonist	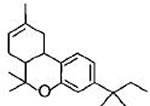	Analgesic, antiemetic, appetite stimulant tumour growth inhibitor [159]
HU-308 CB2 selective agonist	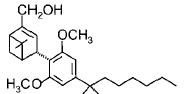	Tumour growth inhibitor (in glioma, skin carcinoma, lymphoma [159]
CP55940 CB/CB2 agonist	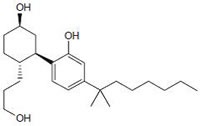	Analgesic, antiemetic, appetite stimulant, tumour growth inhibitor, multiple sclerosis [159]
*R*-(+)-methanandamide CB1 agonist	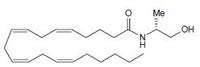	Analgesic, antiemetic, appetite stimulant tumour growth inhibitor [159]
AM251 CB1 antagonist	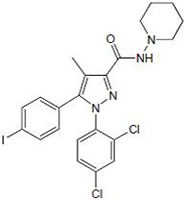	Metabolic syndrome [180]
AM281 CB1 antagonist	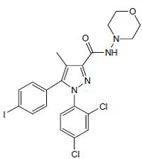	Improves recognition loss induced by naloxone in morphine withdrawal mice, various pharmacological property [181-182]

### Endogenous cannabinoids

Endogenous cannabinoids which are produced in our body include lipid molecules containing long-chain polyunsaturated fatty acids, amides, esters and ethers that bind to CB1 or CB2 receptors. Several pharmacological evidences show that endocannabinoids also exert biological effects through non-CB1/CB2 receptors [13]. Endocannabinoids mainly act as neuromodulators or retrograde messengers which affect the release of various neurotransmitters in the peripheral and neural tissues [14]. They also play important role in inflammation, insulin sensitivity, and fat and energy metabolism. Inhibition of endocannabinoids may be a tool in reducing the prevalence of metabolic syndrome [15]. Two of the best characterized endocannabinoids are N-arachidonoylethanolamine (AEA-anandamide) and 2- arachidonoylglycerol (2-AG) which affect our mood, appetite, pain sensation, inflammation response, and memory [7, 16]. Anandamide which was isolated from porcine brain in 1992 was shown as the first brain metabolite, to function as a ligand for CB1, [7]. 2-arachidonoyl-glycerol which was isolated from canine gut acts through both CB1 and CB2 receptors [16-17]. Palmitoyl-ethanolamide, or N-(2-Hydroxyethyl) hexadecamide (N-acyl-ethanolamide) is co-synthesized with anandamide in all tissues and acts through CB2 [18-20]. Other unsaturated fatty acid ethanolamides like Docosatetraenylethanolamide and Homo-γ-linoenylethanolamide act as agonists for the neuronal CB1 receptor [20-21]. Another putative endogenous cannabinoid, oleamide, or cis-9-octadecenoamide, has also been isolated and shown to have similar actions to anandamide in the behavioral rodent tests [22].

### Phytocannabinoids

Phytocannabinoids are only known to occur naturally in significant quantity in the cannabis plant, and are concentrated in a viscous resin that is produced in glandular structures known as trichomes. Δ^9^-THC, cannabidiol (CBD) and cannabinol (CBN) are the most prevalent natural cannabinoids [23]. Δ^9^-THC binds with similar affinities for both CB1 and CB2 receptors at submicromolar concentration. It behaves as a CB1 receptor partial agonist and CB1/CB2 receptor antagonist [24]. Δ^8^-THC has similar affinities for CB1 and CB2 receptors as like Δ^9^-THC [25]. Other common cannabinoids are cannabidiol (CBD), Cannabigerol (CBG), Cannabichromene (CBC), Cannabicyclol (CBL), Cannabivarin (CBV), Tetrahydrocannabivarin (THCV), Cannabidivarin (CBDV), Cannabichromevarin (CBCV), Cannabigerovarin (CBGV), Cannabigerol Monoethyl Ether (CBGM).

### Synthetic cannabinoids

Synthetic cannabinoids have been extensively used as a pharmacological agent, both *in vitro* and *in vivo*, to obtain more detailed insight of cannabinoid action, in order to evaluate their potential clinical use. They showed both antineoplastic and protumoral activity, depending on type of agonist, target tissues, route of administration, doses and duration of the treatment [26-27]. Synthetic cannabinoids are classified on the basis of chemical structure of molecules and they are capable of a more selective activation of cannabinoid receptor [28].

#### a) Classical cannabinoids

Compounds isolated from the plant C. sativa or synthetic analogs of these compounds fall into this category. HU-210, Δ^9^-THC, Δ^8^-THC and desacetyl-L-nantradol are synthetic cannabinoids which behave as CB1/CB2 receptor agonists (lack of CB1/CB2 selectivity. The most psychotropic component of the C.sativa plant is Δ^9^-THC which shows affinity for both the cannabinoid receptors. Increased affinity of HU-210 is due to replacing pentyl side chain of Δ^8^-THC with a dimethylheptyl group. Other CB2-selective agonists that have been synthesized by structurally modifying THC molecule are JWH-133, JWH-139, and HU-308 and L-759633 and L-759656 which was effective in nanomolar range [29-31].

#### b) Nonclassical Cannabinoids

These are a family of AC-bicyclic and ACD-tricyclic cannabinoid analogs. Furthermore bi-cyclic analog, CP55940, an important cannabinoid agonist has similar affinity for CB1 and CB2 receptors. Also, it is highly potent *in vivo*. CP55244 and CP47497 are other cannabinoids that fall in this category.

#### c) Aminoalkylindoles

These are a family of aminoalkylindoles with cannabimimetic properties. R-(+)-WIN55212 is the most well known compound in this series. It exhibits high affinity for both cannabinoid receptors, but more selective for CB2. It has similar pharmacological effects like THC *in vivo*. JWH-015 and L-768242 also show affinity towards CB2 than R-(+)-WIN55212 [32].

#### d) Eicosanoids

Anandamide, which is an endogenous cannabinoid ligand was originally discovered in mammalian brain and other tissues and acts similar to THC. Methanandamide, its R-(+)-isomer is nine times more CB1 specific than the S-(+)-isomer [33]. 2-arachidonoylglycerol, another well studied endocannabinoid has both CB1 and CB2 affinities. Other compounds are arachidonyl-2-chloroethylamide (ACEA) and arachidonylcyclopropylamide (ACPA).

#### e) Others

These represent diarylpyrazole compounds which function antagonistic to cannabinoid receptors [34]. SR141716A is a potent CB1 antagonist and SR144528 is a CB2 antagonist [35-36]. AM251 and AM281 are analogs of SR141716A which block CB1 receptor-mediated effects.

### Cannabinoid receptor mediated signaling in cancer

**Fig.2:**
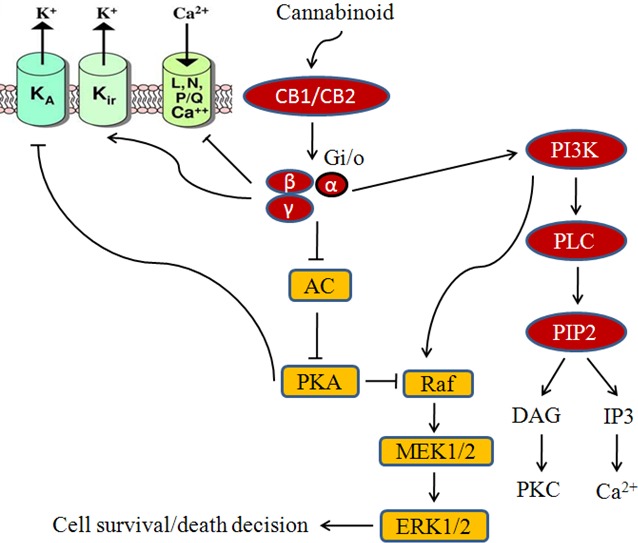
Cannabinoid mediated signaling in cancer cells Cannabinoids activate CB1 or CB2 receptor which in turn modulates diverse signaling targets.

The widespread distribution of cannabinoid receptors (CB1/2,TRPV1) regulate a variety of central and peripheral physiological functions, including neuronal development, neuromodulatory processes, energy metabolism as well as cardiovascular, respiratory, reproductive functions. CB1/2 receptors are also responsible for proliferation, motility, invasion, adhesion and apoptosis of cancer cells both *in vitro* and *in vivo* (Table II). CB1/2 receptor activation leads to various events like affecting Ca2^+^ and K^+^ channels, modulation of adenyl cyclase and cyclic AMP (c-AMP) levels in most tissues and models, regulation of members mitogen activated protein kinase family (MAPKs), like extracellular signal regulated kinase-1 and -2 (ERK1/2), p38, MAPK and c-Jun N terminal kinase (JNK) [37-38] as shown in Fig.2.

**Table II:** Role of cannabinoid in different cancers and its associated signaling

Cannabinoids	Anti-cancer effect and its mechanism of action
Anandamide	1)Breast cancer: (blocks G1 - S phase transition)-Regulates Raf- 1/ERK/MAP pathway, Wnt/β catenin signaling 2)Prostate cancer: Regulates EGFR pathway
THC	1)Breast cancer: (block G2 - M phase transition)- Activates the transcription factor JunD Anti-tumor action in MMTV-neu mice via inhibition of AKT Anti-invasive effect-modulate MMP-2/MMP-9 pathway 2)Prostate cancer:PI-3/AKT and Raf-1/ERK1/2 pathway Mitogenic effect at low doses. 3)Lung cancer: ERK1/2, JNK and AKT pathway Mitogenic effect at low doses. 4)Glioma: MMP-2pathway, ER stress mediated autophagy 5)Lymphoma: MAPK/ERK pathway
2-AG	1)Breast cancer: Suppression of nerve growth factor Trk receptors and prolactin receptors Prostate cancer: NF-κB/cyclin D and cyclin E, Suppression of nerve growth factor Trk receptors and prolactin receptors. 2)Glioma: Inhibition Ca(2+) influx 3)Bone cancer: Attenuates mechanical hyperalgesia
HU120	1)Prostate cancer: AKT pathway
WIN-55,212-2	1)Breast cancer: Regulates COX-2/PGE2 signaling pathway 2)Prostate cancer: Sustained activation of ERK1/2 3)Skin cancer: Inhibits pro-angiogenic growth factor, AKT and pRB pathway 4)Glioma: Ceramide and NF-Κb pathway 5)Lymphoma: Ceramide and p38 pathway
R-(+)-MET	1)Breast cancer: decreased phosphorylation of focal adhesion–associated protein kinase and Src and tyrosine kinases involved in migration and adhesion 2)Prostate cancer: mitogenic effect at low dose
JWH-133	1)Breast cancer: inhibition of AKT-Regulate COX-2/PGE2 signaling pathway 2)Lung cancer: MMPs pathway 3)Skin cancer: G1 arrest-AKT pathway
Met-F-AEA	1)Breast cancer: S phase cell cycle arrest Regulates FAK/Src and RhoA-ROCK pathways
JWH-015	1)Breast cancer: CXCR-4/CXCL12 pathway 2)Prostate cancer: JNK/AKT signaling pathway
Δ^9^-THC	1)Breast cancer: mitogenic effect in cells expressing low levels of CB1/CB2 receptors. 2)Prostate cancer: PI3K/Akt and Raf-1/ERK1/2 pathway Mitogenic at low doses 3)Lung cancer: EGFR/ERK1/2, c-Jun-NH2-kinase1/2, and Akt pathway. Mitogenic at low doses 4)Glioma: MMP-2 pathway
CBD	1)Breast cancer: ER stress/ERK and reactive oxygen species (ROS) pathways 2)Prostate cancer: ERK1/2 and AKT pathways 3)Lung cancer: up-regulation of TIMP-1 Cox-2 and PPAR-γ regulation 4)Cervical cancer: Up-regulation of TIMP1
CBDA	1)Breast cancer: PKA/RhoA pathway
AME1241	1)Bone cancer: Anti-nociception

### 1. Role of cannabinoids in regulation of cancer growth

One of the important aspects of an effective anti-tumor drug is its ability to inhibit proliferation of cancer cells. Cancer cells proliferate rapidly in uncontrolled manner. Also, these cells escape death mechanism which a normal cell undergoes like apoptosis. Apoptosis is a kind of programmed cell death (PCD) mechanism which involves activation of caspase dependent and independent pathways [39]. Cannabinoids have been proved to be anti-proliferative and apoptotic drugs. This section comprises of the detailed role of cannabinoids in modulation of tumor proliferation, cell cycle and apoptosis in various cancer types.

### Cannabinoids and breast cancer

Breast cancer is one of the most common human malignancies and the second leading cause of cancer-related deaths in women, and its incidence in the developing world is on the rise [40-41]. It represents approximately 30% of newly diagnosed cancers each year. It is mainly classified into three main subtypes according to their molecular profiles: hormone receptor-positive, HER2-positive (ErbB2-positive, a member of EGFR family) and triple-negative tumors [42-43]. Cannabinoid-based medicines have been useful for the treatment of these three breast cancer subtypes.

CB1 and CB2 receptor expression has been described in different breast cancer tissue and cell line by immunohistochemistry, RT-PCR and western blot. CB1 expression was detected in 14% of human Her-2 positive breast cancer tumor tissue and 28% of human breast carcinoma [26, 44]. But no correlation between CB1 expression and ErbB2 expression was found [44]. CB1 receptors are also present in different breast cancer cell lines (MCF-7, T-47D, MDA-MB-231, TSA-E1, MDA-MB-468) and in human breast tissues [26, 45-51]. By contrast, CB2 immunoreactivity was detected in 72% of human breast tumor tissue and 91% of ErbB2-positive tumor tissue, suggesting a link between CB2 and ErbB2-expression [44]. The expression of CB2 receptor was also analyzed in different breast carcinoma cell lines (MCF-7, T-47D, MDA-MB-231, MDA-MB-468, EVSA-T, SkBr3) and human breast tissues [26, 44-46, 49, 51-52]. The putative novel cannabinoid receptor subtype GPR55 was highly expressed in a MDA-MB-231 cells, but it is expressed at lower (30-fold) levels in MCF-7 cells [53].

**Fig.3:**
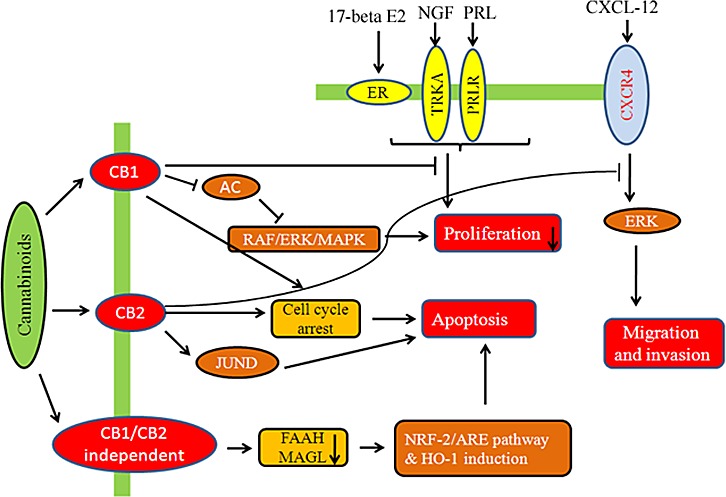
Modulatory effect of cannabinoids on hormone sensitive breast cancer cells Cannabinoids are involved in receptor dependent/independent regulation of various hallmarks of breast cancer like proliferation, migration, invasion, etc.

**Fig.4:**
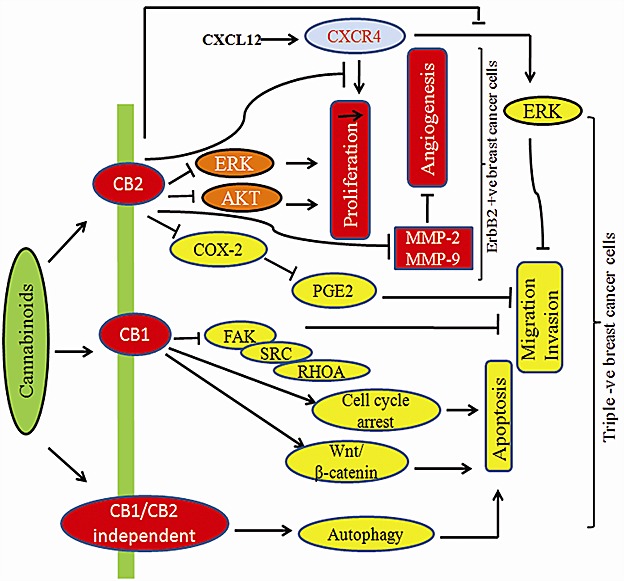
Modulatory effect of cannabinoids on HER-2 +ve and Triple −ve breast cancer cells Cannabinoids inhibit key signaling targets in triple negative breast cancer which has worse prognosis in patients.

Cannabinoids modulate the growth of hormone sensitive breast cancer cells as shown in Fig.3 and 4. JWH-O15 inhibits hormone sensitive breast cancer metastasis by modulating CXCL12/CXCR4 signaling axis [27, 54]. Endocannabinoids such as anandamide (AEA) are important lipid ligands regulating cell proliferation, differentiation and apoptosis. Their levels are regulated by hydrolase enzymes, the fatty acid amide hydrolase (FAAH) and monoacylglycerol lipase (MGL). Breast tumor cells express FAAH abundantly. Inhibition of FAAH (siRNA-FAAH or FAAH inhibitor URB597) induced cell death by activating nuclear factor (erythroid-derived 2)-like 2 (Nrf2)/antioxidant responsive element (ARE) pathway and heme oxygenase-1 (HO-1) induction and transcription [55]. Anandamide inhibits basal and nerve growth factor (NGF) induced proliferation of MCF-7 and EFM-19 cells in culture through CB1 receptor and Δ^9^-THC inhibits 17beta-estradiol-induced proliferation of MCF7 and MCF7-AR1 cells [45, 56-58]. Δ^9^-THC also inhibits cell proliferation of ER^−^/PR^+^ breast cancer cells. The effects of anandamide and Δ^9^-THC were mediated by blocking transition from one phase of cell cycle to another, G1-S and G2-M respectively [49, 56, 59-60]. Cell cycle arrest is responsible for apoptotic cell death. The analog of anandamide, Met-F-AEA reduces MDA-MB-231 proliferation by arresting cells in the S phase of the cell cycle [60]. Anandamide inhibits adenylyl cyclase (AC) and thus activating the Raf-1/ERK/MAP pathway in ER^+^/PR^+^ breast cancer cells whilst THC activates the transcription factor JunD to finally execute action towards apoptosis in ER^−^/PR^+^ breast cancer cells [49, 56, 59]. One study shows that anandamide inhibits proliferation of MDA-MB31 cells by modulating Wnt/β-catenin signaling pathway [61]. This effect is occurred by inhibition of the cyclin-dependent kinase CDK2 [50, 60].

Synthetic cannabinoids containing naphthoylindole, JWH-018, JWH-073, JWH-122 and JWH-210 and of one benzoylindole AM-694 shows anti-estrogenic property in MCF-7 cells [62]. Cannabinoid induced signaling in ER^+^ breast cancer cells is shown in Fig.3. WIN 55,212-2 and JWH-133 also produce an inhibition of MDA-MB-231 proliferation by blocking the progression trough the cell cycle, G1 to S phase transition and induced apoptosis [26]. The anti-proliferative effect of WIN 55,212-2 and JWH-133 is validated in both in xenograft-based and PyMT genetically engineered model of triple-negative breast cancer modulate through the COX-2/PGE2 signaling pathway [26]. JWH-015 also reduces breast cancer-induced bone pain, bone loss, and breast cancer proliferation via cytokine/chemokine suppression.

CBD inhibits AKT and mTOR signaling as well as decreased levels of phosphorylated mTOR and 4EBP1, and cyclin D1. CBD enhances the interaction between beclin1 and Vps34; it inhibits the association between beclin1 and Bcl-2 [63]. LPI stimulates proliferation and this effect was blocked by CBD [53]. Thus, cannabinoids along with COX-2 inhibitors or other chemotherapeutic agents may represent as novel chemopreventive tools for the treatment of breast cancer. Fig.4 shows the effect of cannabinoids on HER-2 and triple negative breast cancer pathway.

### Cannabinoids and prostate cancer

Prostate cancer is the most common malignancy among men of all races and is one of the leading causes of cancer death in this population. CB1 and CB2 expression levels were higher in prostate cancer tissues and several cell lines including PC-3, DU-145, LNCaP, CWR22Rv1, CA-HPV-10 as compared with normal prostate epithelial cells [45, 64-71]. Moreover, the putative cannabinoid receptor GPR55 is also expressed in PC-3 and DU-145 cells [72]. Δ^9^ –THC, WIN-55,212-2, R(+)-Methanandamide, Cannabidiol (CBD), Anandamide, JWH-015, HU120, 2-AG and its stable analogue noladin have exerted anti-proliferative, apoptotic and anti-invasive effects in different prostate cancer cells both *in vitro* and *in vivo* [45, 68-69, 73-77]. Δ^9^ –THC induced apoptosis via a receptor-independent manner whilst in another study, the same group reported that activation of cannabinoid receptors in PC-3 cells stimulated the PI3K/Akt pathway with sequential involvement of Raf-1/ERK1/2 and nerve growth factor induction [66, 68]. Treatment of WIN-55,212-2 resulted in sustained activation of ERK1/2 and inhibition of AKT, which was associated with the induction of phosphatases [74, 78]. CBD also mimicked the same effect in LNCaP cells as WIN-55,212-2 [78]. The effects of anandamide in LNCaP, DU145, and PC3 cells were mediated through down-regulation of epidermal growth factor receptor (EGFR) and accumulation of ceramide [77]. JWH-015 triggered a de novo synthesis of ceramide, which induced cell death, followed by JNK (c-Jun N-terminal kinase) activation and Akt inhibition [76]. Effects of R(+)-Methanandamide and JWH-015 were rescued by treatment with SR 144528 in PC-3 cells [76, 79]. Interestingly, (R)-methanandamide was shown to have a mitogenic effect on LNCaP cells at very low doses [46, 75]. FAAH is a serine hydrolase that metabolizes N-acylethanolamines including AEA, OEA and PEA to fatty acids plus ethanolamine. A recent report showed that FAAH is also over-expressed in prostate cancer cells and the inhibition of FAAH can enhance the survival of cancer patient [80-81].

### Cannabinoids and lung cancer

Lung cancer has one of the highest mortality rates among cancer-suffering patients. Cannabinoids (CBs) could halt tumor development without side effects via specific targeting of CB1/CB2 receptor. Studies suggest the involvement of COX-2 and PPAR-γ in CBD's proapoptotic and tumor-regressive action in A549, H460 cells and primary cells from a patient with lung cancer [82]. Moreover, CBD caused up-regulation of COX-2 and PPAR-γ in tumor tissue and tumor regression in A549-xenografted nude mice [82]. JWH-133 induced anti-proliferative potential in A549 cell line via DNA fragmentation [83]. Recently, we published results on the role of FAAH in regulating the effects of AEA in NSCLC. We showed that blocking FAAH increases the levels of AEA, which in turn inhibits EGFR signaling pathway, ultimately leading to cell cycle arrest and apoptosis [84]. These results generate a rationale for further *in vivo* efficacy studies with this compound in preclinical cancer models.

### Cannabinoids and skin cancer

Melanoma is the mainly cause of skin cancer–related deaths worldwide. CB1 and CB2 receptors are expressed in normal skin and skin tumors of mice and humans [85]. Activation of CB1/CB2 receptors induced the apoptotic death of tumorigenic epidermal cells, without affecting the nontransformed epidermal cells. WIN-55,212-2 or JWH-133 induced anti-proliferative effect in epidermal cell lines (PDV.C57 and HaCa4) and reduces malignant tumors in nude mice [85]. WIN-55,212-2 or JWH-133 induced G1 cell cycle arrest on melanoma cells, via inhibition of p-Akt and hypophosphorylation of the pRb retinoblastoma protein tumor suppressor [85].

### Cannabinoids and pancreatic cancer

Pancreatic cancer is one of the most aggressive and devastating human malignancies. CB1 and CB2 receptors were expressed in normal and pancreatic cancer tissues, analyzed by RT-PCR [86]. Cannabinoid receptors on pancreatic cancer cells may affect prognosis and pain status of PDAC patients [86]. Cannabinoid administration leads to apoptosis of pancreatic tumor cells via CB2 receptor and ceramide-dependent up-regulation of p8 and ATF-4 and TRB3 stress–related genes [87]. Another study showed that CB1 receptor antagonist AM251–induced cell death in pancreatic MIAPaCa-2 cells occurred via receptor-independent manner [88].

### Cannabinoids and bone cancer

Chondrosarcoma and osteosarcoma are the most frequent primary bone cancers [89]. Bone metastases are a frequent complication of cancer and the most frequent type of pain related to cancer. Breast cancer and prostate cancer mainly metastasize to bone which act as a fertile soil for the growth of secondary tumors [90]. The skeletal endocannabinoid system plays a significant role in regulating bone mass and bone turnover. The expression levels of CB1 and CB2 receptors were analyzed in bone cancer patient using immunohistochemistry [91]. Bone metastatic patient has severe pain so cannabinoids can attenuate pain and hyperalgesia [92]. Sativex is the combination of delta-9-tetra-hydrocannabinol and cannabidiol, used to treat pain in cancer [92]. WIN55,212-2 induces apoptosis in the NCTC-2472 sarcoma cell line and AM1241 produced a reduction in bone loss in bone tumor animal model (NCTC-2472 cell line injected in to femur of mice) [93-94]. Effects of subcutaneously administered WIN55,212-2 on weight bearing and mechanical hyperalgesia were consistent with cannabinoid receptor mediated anti-nociception [93]. WIN55,212-2 also attenuates tumour-evoked mechanical hyperalgesia following local (intraplantar) administration through activation of CB1 and CB2 receptors [95]. Injection of CP55 940 produced anti-nociceptive properties in the tail flick test and suppressed mechanical hyperalgesia in NCTC-2472 or melanoma B16-F10 xenografted bone tumor model [96]. Indeed, intraplantar administration of AEA reduces mechanical hyperalgesia, URB597 increases AEA levels and decreases hyperalgesia in a model of calcaneous bone cancer pain [91]. However, intrathecal administration of either URB597 or MGL (URB602) inhibitors failed to produce anti-nociception when tested for spontaneous flinches, limb use and weight bearing [97]. Moreover, the CB1 agonist arachidonoyl-2-chloroethylamide (ACEA) produces anti–nociceptive properties following intrathecal administration in this model; ACEA suppressed spontaneous flinches and increased limb use and weight bearing [97]. AM1241 produces significantly reduced bone loss and decreased the incidence of cancer-induced bone fractures [94, 98]. Administration of JWH-015 and AM1241 attenuated tumor-evoked tactile allodynia and thermal hyperalgesia by reducing NR2B-dependent activity [98-99]. CB2 agonist, JWH-015 reduced breast cancer induced bone pain, bone loss, and breast cancer cell proliferation via cytokine/chemokine suppression in murine mammary cell line implanted into the femur intramedullary space [100]. JWH-015 increased survival without the major side effects of current therapeutic options.

### Cannabinoids and glioma

Gliomas are the most important group of malignant primary brain tumors and one of the most aggressive forms of cancer, exhibit high resistance to conventional chemotherapies. In glioblastoma endothelial cells, CB1 and CB2 receptors were present in about 38% and 54% of the cells respectively, analyzed by immunohistochemistry. CB2 expression levels were higher in glioblastoma tissues in comparison to CB1. Selective CB2 agonists may become important targets for the treatment of glioma. Cannabinoids, inhibit tumor growth in animal models by inducing apoptosis of tumor cells and impairing tumor angiogenesis. Administration of Δ^9^-THC and JWH-133 inhibits MMP-2 expression in *in vivo* model of glioma [101-103]. The growth inhibitory effect of these cannabinoids is prevented by blocking ceramide synthesis, and the expression of the stress protein p8 [102-103]. Both Δ^9^-THC and WIN-55,212-2 resulted in sustained activation of ERK1/2 and inhibition of AKT [104]. Furthermore, Δ^9^-THC induced eukaryotic translation initiation factor 2alpha (eIF2alpha) phosphorylation and thereby activated an ER stress response that promoted autophagy via tribbles homolog 3-dependent (TRB3-dependent) inhibition of the Akt/mammalian target of rapamycin complex 1 (mTORC1) axis [105]. The activation of this pathway was necessary for the antitumor action of cannabinoids *in vivo* [105]. In contrast to that CBD treatment induces apoptosis in glioma cells *in vitro* and tumor regression *in vivo* through activation of caspases and reactive oxygen species via receptor-independent manner Furthermore, studies revealed that CBD induced TRPV2-dependent Ca^2+^ influx which triggers the drug uptake and synergizes with cytotoxic agents to induce apoptosis of glioma cells [106]. Authors thought that CBD which do not specifically interact with CB1/CB2 receptors, can modulate the activity of Δ^9^-THC. On that basis Marcu et al determined the growth inhibitory effect of CBD in combination with Δ^9^-THC in the U251 and SF126 glioblastoma cell lines [107]. Furthermore, the combined treatment of Δ^9^-THC and temozolomide (TMZ) exert a strong antitumoral action in glioma xenografts by inducing autophagy [108]. The submaximal doses of Δ^9^-THC and CBD in combination with TMZ produced a strong antitumoral action in both TMZ-sensitive and TMZ-resistant tumors [108]. Treatment of KM-233 (novel cannabinoid ligand) caused a time dependent change in the phosphorylation profiles of MEK, ERK1/2, Akt, BAD, STAT3, and p70S6K in U87MG human GBM cells [109]. At 12mg/kg daily dose of KM-233 for 20 days revealed around 80% reduction in tumor size in the orthotopic model of U87MG [109]. Glioma cells develop resistance to cannabinoid treatment due to the upregulation of Amphiregulin (EGFR family ligand) and the growth factor midkine (Mdk) [110-111]. Amphiregulin expression was associated with increased ERK activation and Mdk mediated its protective effect through ALK which interferes with autophagic cell death [112]. The silencing of amphiregulin and Mdk or ALK pharmacological inhibition can overcome drug resistance of glioma to cannabinoids antitumoral action. Furthermore, to improve the efficacy of cannabinoids action, microencapsulation methods were used which facilitates a sustained release of the two cannabinoids for several days [113]. Administration of CBD- and THC-loaded poly-ε-caprolactone microparticles reduced tumor growth, cell proliferation and increased apoptosis in mice bearing glioma xenografts with the same efficacy than a daily local administration of these drugs in solution [113].

### Cannabinoids and lymphoma

CB1 and CB2 receptors were over-expressed in mantle cell lymphoma (MCL), and B cell non-Hodgkin lymphoma [114-115]. Δ^9^-THC inhibits cell viability and increased apoptosis both in vitro in EL4 and MCL cells and EL4 tumor bearing mice. In next studies the combination of Δ^9^-THC and other cytotoxic agents induced apoptosis in leukemia cells by MAPK/ERK pathway [114, 116]. In addition R(+)-methanandamide and WIN-55,212-2 induced apoptosis in MCL cells, was associated with ceramide accumulation and p38, depolarization of the mitochondrial membrane, and caspase activation [117]. R(+)-methanandamide also induced apoptosis in CLL cells [118]. In contrast, cannabinoids decreased cell viability as assessed by metabolic activity. The persistent expression of mammalian homolog of Atg8 with microtubule-associated protein-1 light chain-3 II (LC3 II) and p62, as well as the lack of protection from chloroquine, indicates that lysosomal degradation is not involved in this cytoplasmic vacuolation process, distinguishing from classical autophagy [119]. Paraptosis-like cell death-a third type of a programmed cell death occurred in response to cannabinoids [119].

### Cannabinoids and oral cancer

Oral cancer is mainly occurs in the mouth including lips, tongue and throat. Smoking, tobacco chewing and alcohol consumption increases the incidence of oral cancer. Radiation therapy and surgery is the common treatment for oral cancer. Δ^9^-THC induced apoptosis in oral squamous cell carcinoma (OSCC), a malignant form of oral cancer [120].

### Cannabinoids and head and neck cancer

Marijuana smoking increases the incidence of head and neck cancer in young people but its constituent, cannabinoids have anti-tumor properties. One study reports that moderate marijuana use is associated with reduced risk of HNSCC [121].

### Cannabinoids and thyroid carcinoma

Thyroid carcinoma is the most aggressive form which occurs in thyroid gland. IL-12 gene transfer in to anaplastic thyroid carcinoma cell line (ARO) has anti-tumorigenic effect [122]. This effect was observed due to the activation of cannabinoid receptor. Furthermore they have reported that CB2 agonist JWH-133 and CB1/CB2 agonist WIN-55,212-2 induced apoptosis in ARO and ARO/IL-12 cells [122]. 2-methyl-2'-F-anandamide (Met-F-AEA) also induced apoptosis in thyroid carcinoma cells via activation of p53 and p21 mediated pathway [123].

### 2. Role of cannabinoids in pro-metastatic mechanisms like angiogenesis, migration and invasion

Migration and invasion are characteristic features of cancer cells. Carcinoma cells that are invasive have higher migratory potential which helps them to disseminate into the surrounding tissues and spread to other organs, ultimately leading to metastasis [124]. Angiogenesis, which involves growth of new vasculature has been shown to be closely related to cancer metastasis. Developing novel anti-invasive and anti-angiogenic targets would be more effective in inhibiting metastasis at earlier stage [125].

In breast cancer, Met-F-AEA leads to the inhibition of the focal adhesion kinase (FAK) and RhoA-ROCK pathways [126]. JWH-015 reduces CXCL12-induced cell migration and invasion of a highly metastatic MDA-MB-231-derived cell line by inhibiting ERK and cytoskeletal focal adhesion and stress fiber formation [127]. Novel synthetic hexahydrocannabinol analogs, LYR-7 and LYR-8 reduced tumor growth by targeting VEGF-mediated angiogenesis signaling in MCF-7 and MCF-7 Tam resistant cells [128]. Cannabidiolic acid (CBDA) inhibits migration of MDA-MB-231 cells via inhibition of cAMP-dependent protein kinase A, coupled with an activation of the small GTPase, RhoA [129]. Lysophosphatidylinositol (LPI), the putative endogenous ligand for GPR55, also stimulates cell migration and invasion in a MDA-MB-231 cell line and its effect is blocked by pretreatment with cannabidiol (CBD) [53, 130]. Recent study shows that THC and JWH-133 exert anti-proliferative, pro-apoptotic, anti-angiogenic, and anti-invasive effects in both *in vitro* and *in vivo* models of ErbB-2 breast cancer by modulating AKT, phospho-S6 ribosomal protein, MMP-2 and MMP-9 expression levels shown in Fig.3 [44, 131-132].

In lung cancer, CBD inhibits invasion of A549 cells both *in vitro* and *in vivo* that was accompanied by up-regulation of tissue inhibitor of matrix metalloproteinase-1 (TIMP-1) and decreased expression of plasminogen activator inhibitor-1 (PAI-1) [133-134]. P38 and ERK1/2 were identified as upstream targets for up-regulation of TIMP-1 [134]. But recent report suggests that CBD induced TIMP-1 via upregulation of intercellular adhesion molecule-1 (ICAM-1) [135]. JWH-133 induced anti-angiogenic potential in A549 cell line via inhibition of MMP-2 secretion respectively [83].

In skin cancer, treatment of WIN-55,212-2 or JWH-133 caused impairment of tumor vascularization and decreased expression of proangiogenic factors such as VEGF, placental growth factor, and angiopoietin-2 [85].

In glioma, [136], one study reveals that CBD also inhibits angiogenesis by modulating MMP-2 pathway and Id-1 gene expression in glioblastoma cells [137-138]. Administration of CBD- and THC-loaded poly-ε-caprolactone microparticles reduced tumor growth and angiogenesis in mice bearing glioma xenografts with the same efficacy than a daily local administration of these drugs in solution [113].

### 3. Role of cannabinoids in cancer metastasis

Met-F-AEA, WIN 55,212-2, JWH-133 and JWH-015 inhibit the migration and invasion of MDA-MB231 breast cancer cells to distant sites such as lung [27, 50, 126, 139-140]. CBD inhibits cell proliferation and invasion of 4T1 cells (mammary metastatic cell line) and reduces primary tumor volume as well as lung metastasis in 4T1-xenografted orthotopic model of nude mice [141-142]. This anti-metastatic effect was mediated by downregulation of Id-1 (a basic helix-loop-helix transcription factor inhibitor), ERK and also by inhibiting the ROS pathway. Furthermore, CBD reduced the number of metastatic foci in 4T1- tail vein injected syngenic model. In lung cancer, JWH-015 and Win55,212-2 inhibit *in vitro* chemotaxis, chemoinvasion and *in vivo* tumor growth and lung metastasis via inhibition of AKT, matrix metalloproteinase 9 expression (MMP-9), but the pretreatment of CB1/CB2 selective antagonists, AM251 and AM630 antagonized their effects [143]. Δ^9^-THC inhibits growth of Lewis lung adenocarcinoma via inhibition of DNA synthesis and it suppresses growth and metastasis of A549 and SW-1573 (human lung cancer cell lines) both *in vitro* and *in vivo* due to inhibition of epidermal growth factor–induced phosphorylation of ERK1/2, c-Jun-NH2-kinase1/2 and Akt [144-145].

### 4. Role of cannabinoids in stemness and cancer

Cancer stem cells (CSC) are part of the tumor cell population. Though they might be very less in number, they have the ability to self renew and replicate to produce enormous cancer cell types. CSCs have been shown to be drug resistant with higher invasive and metastatic potential [146]. Studies show that cannabinoid receptors are involved in differentiation of neural progenitors from ectoderm and hematopoietic progenitors from mesoderm. CB1 and CB2 receptor activation modulate proliferation and differentiation of daughter progenitors. Cannabinoids- HU210, WIN55,212-2, AEA and methAEA induced concentration dependent cytotoxicity in P19 embryonal carcinoma (EC) cells [147]. It involved partial regulation by cannabinoid receptors leading to oxidative stress, necrosis coupled with apoptosis. Both CB1 and CB2 receptors are expressed in glioma stem like cells (GSC). HU-210 and JWH-133 helped in neural differentiation of GSC and blocked GSC mediated gliomagenesis [148]. These open further investigation on the function of cannabinoids and the link between stem cell and tumor progression.

### 5. Role of cannabinoids in energy metabolism and cancer

One of the important by-products in energy metabolism is a set of compounds called Reactive Oxygen Species (ROS) which is produced from mitochondria and consists of H_2_O_2_, superoxide O_2_^−^, hydroxyl radical O_2_^−^, etc. Increased ROS production has been associated with triggering of apoptosis [149]. CBD modulates ERK and reactive oxygen species (ROS) pathways, which lead to down-regulation of Id-1 expression. Id-1, an inhibitor of basic helix-loop-helix transcription factors, has recently been shown to be a key regulator of the metastatic potential of breast and additional cancers [141-142]. Arachidonoyl cyclopropamide (ACPA) or GW405833 (GW) induced AMPK mediated autophagy in pancreatic adenocarcinoma cells is strictly related to the inhibition of energy metabolism through a ROS-dependent increase of the AMP/ATP ratio [150]. The combination of cannabinoids and gemcitabine, a nucleoside analogue used in cancer chemotherapy, synergistically inhibit pancreatic adenocarcinoma cell growth by a ROS-mediated autophagy induction without affecting normal fibroblasts [151]. Cannabidiol (CBD)-induced endoplasmic reticulum stress mediated cell death of MDA-MB231 breast cancer cells, with the coexistence of autophagy and apoptosis [63]. Recently one published report shows that Δ^9^-THC and Δ^8^-THC inhibited mitochondrial oxygen consumption rate via receptor independent manner in oral cancer cells [152]. In primary lymphocytes, treatment with CBD induced caspase 8 induced apoptosis which was mediated by oxidative stress. Similar result has been reported in glioma cells where CBD causes oxidative stress and higher enzymatic activities of glutathione reductase and glutathione peroxidase. In NSCLC cell line H460, agonists AEA, THC and HU-210 modulated the activity of mitochondrial complexes I and II-III, decreasing the mitochondrial membrane potential. Δ^9^-THC and cannabidiol acted synergistically to inhibit cell proliferation, modulations of the cell cycle and induction of reactive oxygen species and apoptosis as well as specific modulations of extracellular signal-regulated kinase and caspase activities in glioblastoma [107]. KM-233 induced mitochondrial depolarization, cleaved caspase 3, significant cytoskeletal contractions, and redistribution of the Golgi-endoplasmic reticulum structures in U87MG human GBM cells [109].

### 6. Role of cannabinoids in immune environment and cancer

Cancer is a type of inflammatory disease, where immune cells infiltrate into the tumor site and secrete factors which enhance the prospects of proliferation, angiogenesis and metastasis [153]. Hence, it is important to identify anti-cancer agents that target the immune related cancer environment. In glioma, WIN-55,212-2 caused accumulation of ceramide which is essential for cell death and it also had anti-inflammatory effects [154]. WIN55,212-2, abolished the PGN-activated cell growth which activates a number of inflammatory pathways, including NF-κB (aggravates tumors) and this effect was reversed by CB1 antagonist AM281 but not by the CB2 antagonist, AM630 [155]. Anandamide reduces proliferation and production of cytokines like IL-2, TNF-α and INF-γ in human T lymphocytes by activating CB2 receptor [156]. In astrocytoma and glioblastoma cells, WIN55,212-2 inhibited IL-1 mediated activation of adhesion molecules and chemokines like ICAM-1, VCAM-I and IL-8, which was receptor independent [157]. In murine T cells, cannabinol decreased IL-2 production by inhibiting nuclear factor of activated T-cells (NF-AT) and activator protein-1 (AP-1) [158]. In CD8+ T lymphocytes, JWH-133 downregulated SDF-1 induced m migration in CB2 receptor dependent manner [159].

## CONCLUSIONS AND FUTURE DIRECTIONS

Cannabinoids exert a direct anti-proliferative effect on tumors of different origin. They have been shown to be anti-migratory and anti-invasive and inhibit MMPs which in turn degrade the extra-cellular matrix (ECM), thus affecting metastasis of cancer to the distant organs. Also, cannabinoids modulate other major processes in our body like energy metabolism, inflammation, etc. These data are derived not only from cell culture systems but also from more complex and clinically relevant animal models. Before cannabinoids could be used in clinical trials, there is need to explore more knowledge on several issues such as anti-tumorigenic and anti-metastatic mechanisms as well as which type of cancer patient populations would be more responsive for cannabinoid based therapies. Data presented in this review suggest that cannabinoids derived from different sources regulate differently signaling pathways, modulate different tumor cell types and host physiological system. It is important to understand which of the cannabinoid receptors are expressed and activated in different tumors as each receptor follows a different signaling mechanism. Furthermore, endocannabinoids- AEA and 2-AG are broken down into secondary metabolites like prostaglandin (PGE_2_) and epoxyeicosatetraenoic acid (EE) which enhance tumor growth and metastasis in diverse cancer types. Understanding the exact signaling by which cannabinoids function will eventually lead to targeted clinical approach. Also, the difference in cellular response to cannabinoids in different cancer types might be due to the effect of the tumor environment which involves inflammatory cells, fibroblasts, endothelial cells, macrophages, etc. Thus, there is a need for an integrative understanding of the role of cannabinoids with respect to the tumor and its microenvironment. The diversity of affecting multiple signaling pathways might pave way for developing cannabinoids that selectively obstruct a particular pathway, thus opening avenues for specific targeted treatments.

Moreover, cannabinoids are more specific to cancer cells than normal cells. The administration of single cannabinoids might produce limited relief compared to the administration of crude extract of plant containing multiple cannabinoids, terpenes and flavanoids. Thus, combination of cannabinoids with other chemotherapeutic drugs might provide a potent clinical outcome, reduce toxicity, increase specificity and overcome drug resistance complications. Additional findings in *in vitro* and *in vivo* models are needed to support studies at preclinical setting.
